# Supplementary material for: Identifying the significant drivers of containerized freight rates: From the perspective of dynamic multiscale dependence
Source: PLoS One. 2026 Apr 21;21(4):e0344386. doi: 10.1371/journal.pone.0344386 (PMC13099089; doi:10.1371/journal.pone.0344386)
Supplement: S1 Appendix — (DOCX) [file pone.0344386.s002.docx]

Appendix

A Time series plots for each timescale.


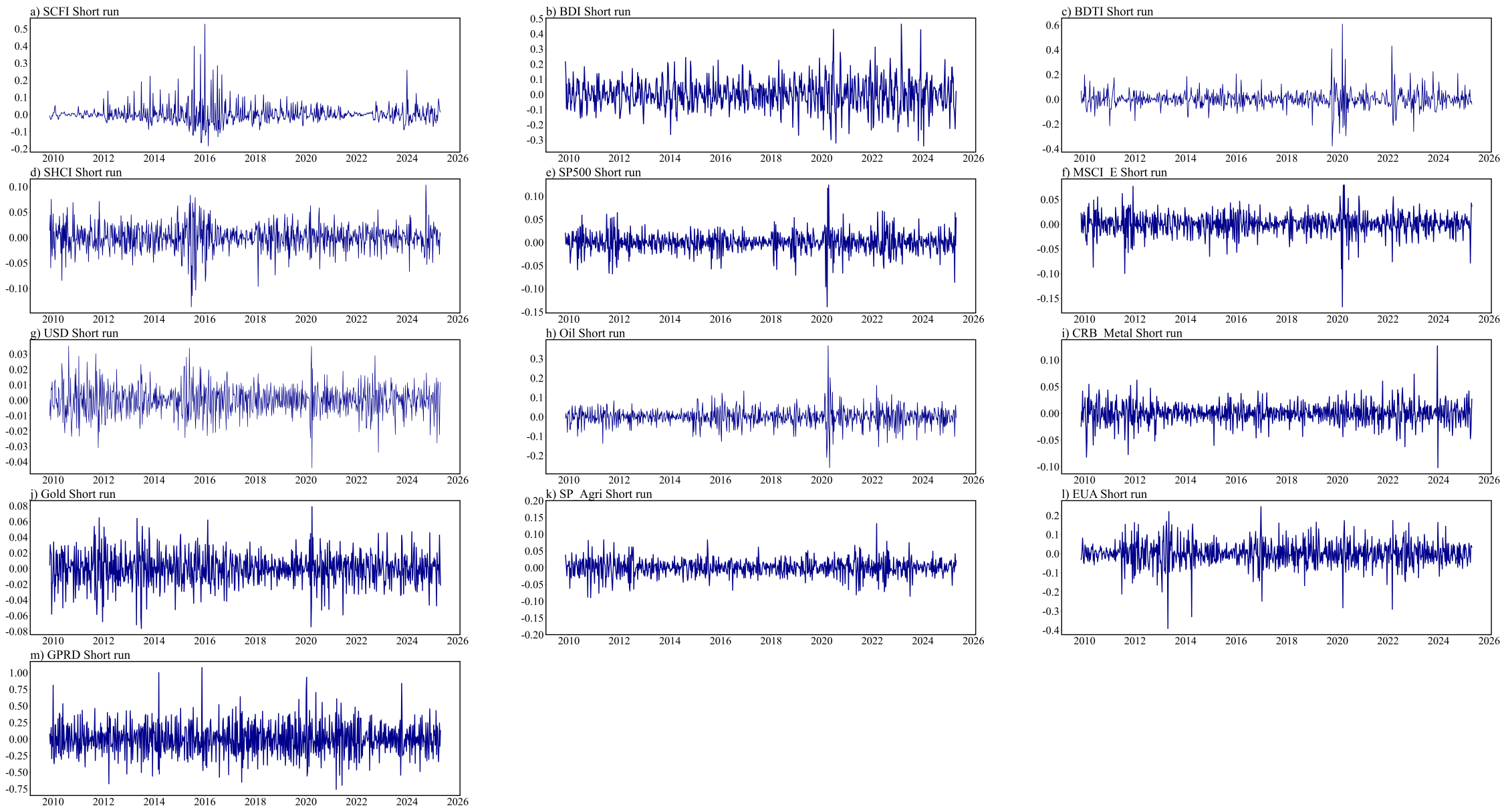


Fig A-1. **Price development for all returns on short scale.**


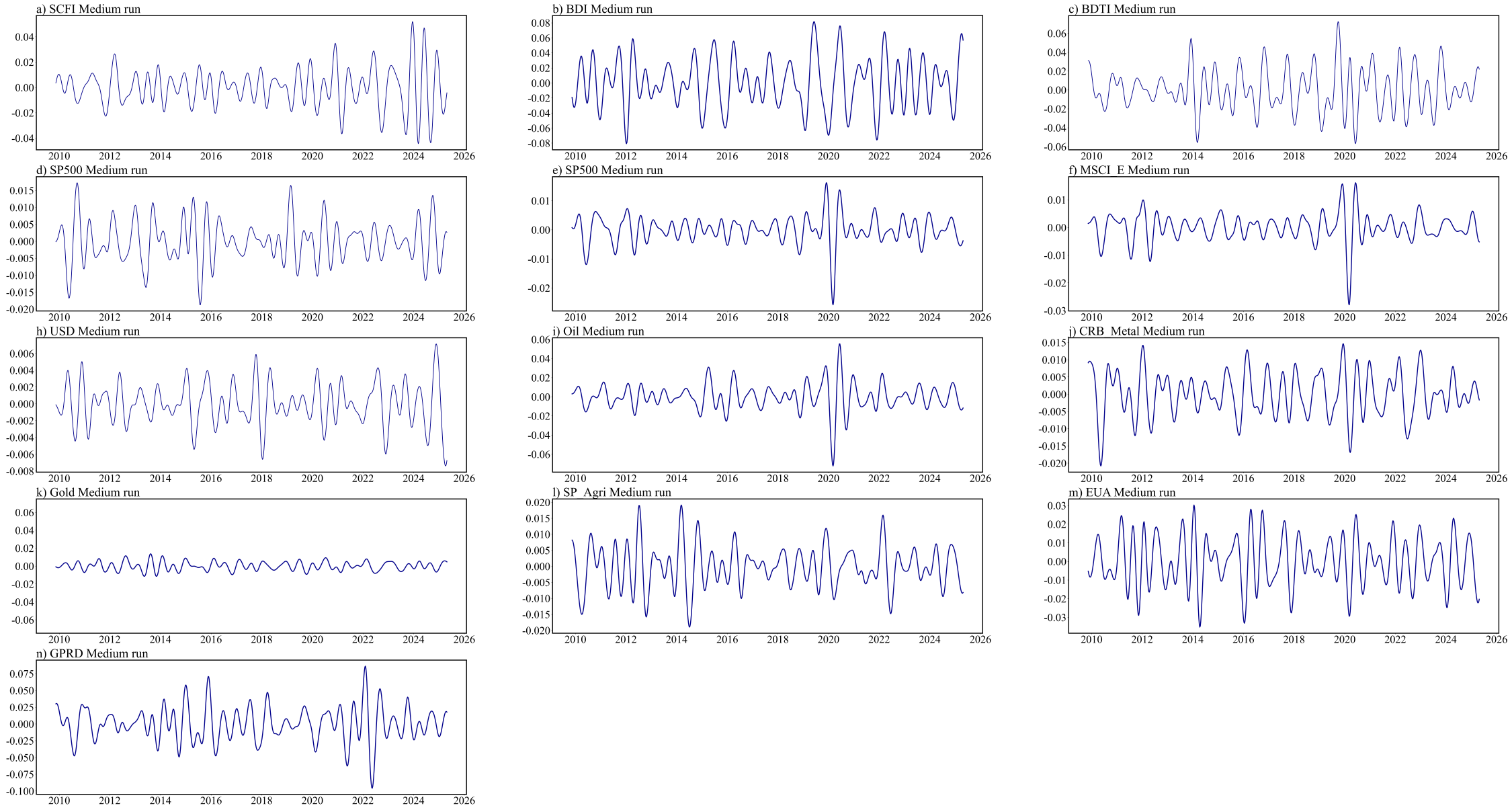


Fig A-2. **Price development for all returns on medium scale.**


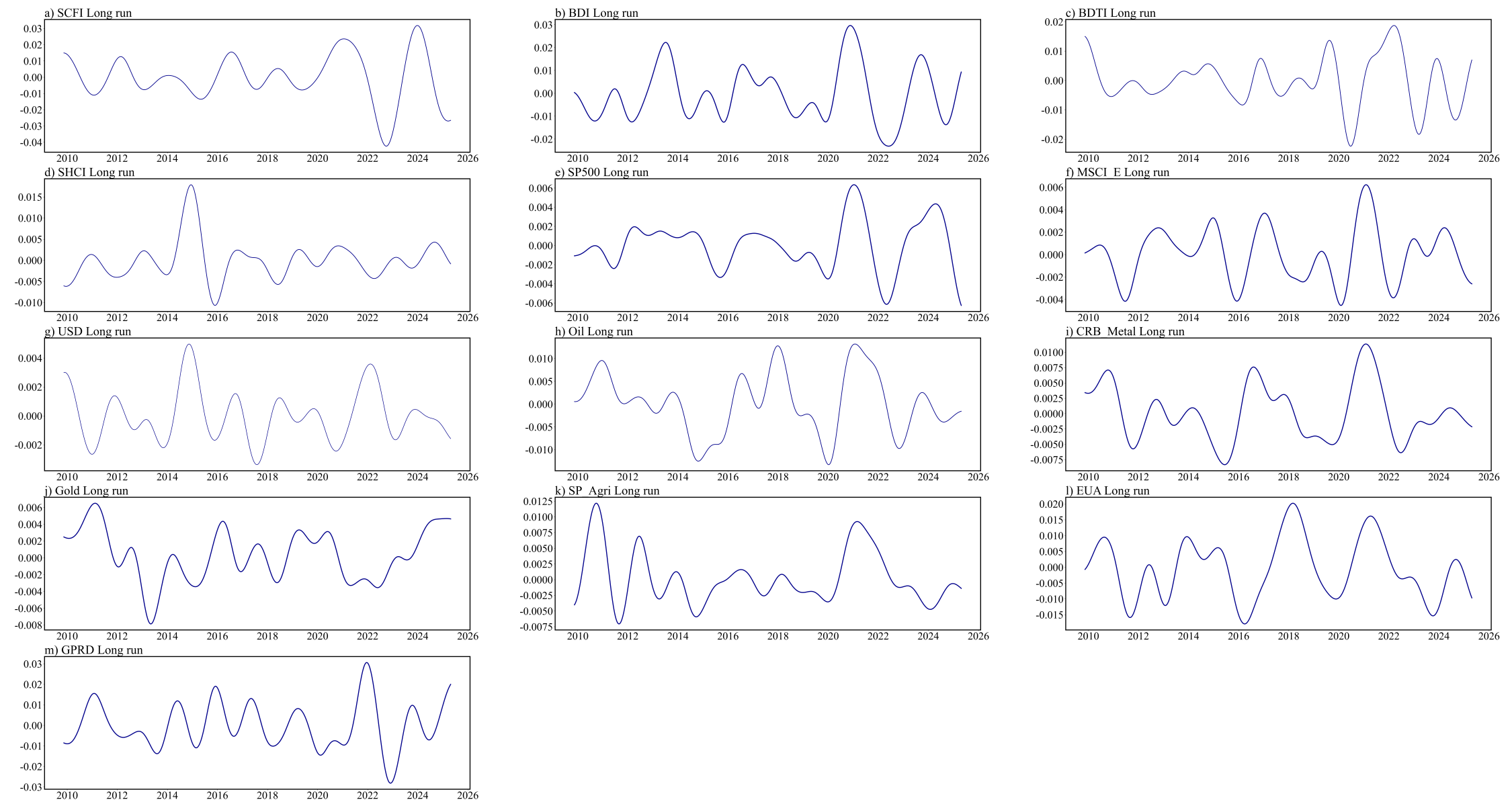


Fig A-3. **Price development for all returns on long scale.**

B Marginal distributions

Table B-1. The marginal distributions for the return series.

| **-** | **BDI** | **BDTI** | **SHCI** | **SP500** | **MSCI_E** | **USD** | **Oil** | **Metal** | **Gold** | **SP_Agri** | **EUA** | **GPRD** | **SCFI** |
| --- | --- | --- | --- | --- | --- | --- | --- | --- | --- | --- | --- | --- | --- |
| **Panel A: Short-term** | | | | | | | | | | | | |  |
| Model | ARMA (3,2)-EGARCH-t | ARMA (5,1)-EGARCH-skewt | ARMA (3,1)-EGARCH-t | - | - | ARMA (2,1)-EGARCH-ged | ARMA (2,4)-EGARCH-t | ARMA (3,2)-EGARCH-t | - | ARMA (0,5)-EGARCH-t | ARMA (2,2)-EGARCH-t | ARMA (1,1)-EGARCH-t | ARMA (4,4)-EGARCH-skewt |
| <<Eqn47>> | -0.1118 | -0.5479** | -0.4095** | - | - | -0.5277** | -0.3366* | -0.1544 | - | -0.6636*** | -0.1750* | -1.1581 | -0.0624 |
| <<Eqn48>> | 0.1340* | 0.4617*** | 0.3711*** | - | - | 0.2168*** | 0.2948*** | 0.1065* | - | 0.3149*** | 0.1958*** | 0.2583** | 0.3542*** |
| <<Eqn49>> | 0.9778** | 0.8984*** | 0.9456*** | - | - | 0.9445*** | 0.9456*** | 0.9804*** | - | 0.9126*** | 0.9683*** | 0.6355** | 0.9875*** |
| <<Eqn50>> | - | 4.1596*** | - | - | - | - | - | - | - |  | - | - | 5.5918*** |
| <<Eqn51>> | - | 0.2573*** | - | - | - | - | - | - | - |  | - | - | 0.3088*** |
| <<Eqn52>> | 17.8672* | - |  | - | - | 1.8173*** | 5.2323*** | 5.5989*** | - | 11.3994*** | 6.0232*** | 12.7245** | - |
| Q(5) p-value | 0.5601 | 0.2188 | 0.2356 | - | - | 0.3626 | 0.0810 | 0.4985 | - | 0.8706 | 0.9632 | 0.5635 | 0 |
| *Q*^2^(5) p-vale | 0.3459 | 0.7403 | 0.2946 | - | - | 0.3235 | 0.0500 | 0.1131 | - | 0.6241 | 0.9882 | 0.8982 | 0.9424 |
| **Panel B: Medium-term** | | | | | | | | | | | | |  |
| Model | ARMA (7,6) | ARMA (4,5) | ARMA (7,6) | ARMA (5,3) | - | ARMA (3,2) | ARMA (5,4) | - | ARMA (5,3) | ARMA (4,5) | ARMA (8,4) | ARMA (4,5) | ARMA (7,7) |
| Q(5) p-value | 1.0000 | 0.9716 | 1.0000 | 0.7409 | - | 0.0288 | 0.1742 | - | 0.6459 | 0.9999 | 1.0000 | 0.9132 | 1.0000 |
| *Q*^2^(5) p-vale | 1.0000 | 1.0000 | 1.0000 | 1.0000 | - | 0.9759 | 1.0000 | - | 1.0000 | 1.0000 | 1.0000 | 1.0000 | 1.0000 |
| **Panel C: Long-term** | | | | | | | | | | | | |  |
| Model | ARMA (7,6) | ARMA (5,3) | ARMA (3,2) | ARMA (10,3) | ARMA (5,2) | ARMA (2,2) | - | - | ARMA (3,6) | ARMA (3,2) | ARMA (4,2) | ARMA (6,4) | ARMA (4,1) |
| Q(5) p-value | 1.0000 | 1.0000 | 0.9999 | 1.0000 | 0.0045 | 1.0000 | - | - | 1.0000 | 0.9763 | 0.9783 | 1.0000 | 0.5199 |
| *Q*^2^(5) p-vale | 1.0000 | 1.0000 | 1.0000 | 1.0000 | 0.9959 | 1.0000 | - | - | 1.0000 | 1.0000 | 0.5732 | 1.0000 | 1.0000 |

Note: Q(5) and *Q*^2^(5) are Ljung-Box test for autocorrelation with 5 lags in residuals and squared residuals. ***, ** and * indicate significance at 1%, 5% and 10% levels respectively.

C The robustness of copula estimations

Table C1. Constant copula estimation results for the short run.

| **-** | **BDI** | **SHCI** | **USD** | **Oil** | **Metal** | **SP_Agri** | **EUA** | **GPRD** |
| --- | --- | --- | --- | --- | --- | --- | --- | --- |
| **Gaussian** | | | | | | | | |
| LL | 0.25 | 0.51 | 0.00 | 0.12 | 1.16 | 1.85 | **0.01** | **0.00** |
| AIC | 1.51 | 0.98 | 2.00 | 1.76 | -0.31 | -1.71 | **1.98** | **2** |
| BIC | 6.20 | 5.67 | 6.69 | 6.45 | 4.38 | 2.99 | **6.68** | **6.69** |
| **Student-t** | | | | | | | | |
| LL | -2.09 | -0.90 | -0.93 | -1.12 | 1.14 | 1.39 | 0.59 | -1.55 |
| AIC | 8.17 | 5.81 | 5.85 | 6.24 | 1.72 | 1.21 | 2.82 | 7.11 |
| BIC | 17.55 | 15.19 | 15.24 | 15.63 | 11.10 | 10.60 | 12.20 | 16.5 |
| **Clayton** | | | | | | | | |
| LL | 0.10 | -0.89 | -0.07 | -1.15 | 0.02 | 0.58 | -2.80 | -1.12 |
| AIC | 1.81 | 3.79 | 2.14 | 4.31 | 1.96 | 0.84 | 7.60 | 4.25 |
| BIC | 6.50 | 8.48 | 6.83 | 9.00 | 6.65 | 5.53 | 12.29 | 8.94 |
| **Rotated Clayton** | | | | | | | | |
| Degree | 180 | 180 | 180 | 180 | 180 | 180 | 180 | 270 |
| LL | **0.89** | **1.15** | **0.10** | **1.12** | **2.72** | **2.54** | -2.80 | -0.76 |
| AIC | **0.21** | **-0.29** | **1.81** | **-0.23** | **-3.45** | **-3.08** | 7.61 | 3.52 |
| BIC | **4.91** | **4.40** | **6.50** | **4.46** | **1.24** | **1.61** | 12.30 | 8.22 |

Note: The log-likelihood, AIC and BIC value are reported. The best-fitted copula is selected based on the AIC, BIC, and log-likelihood value.

Table C2. Constant copula estimation results for the medium run.

| **-** | **BDI** | **BDTI** | **SHCI** | **SP500** | **USD** | **Oil** | **Gold** | **SP_Agri** | **EUA** | **GPRD** |
| --- | --- | --- | --- | --- | --- | --- | --- | --- | --- | --- |
| **Gaussian** | | | | | | | | | | |
| LL | **10.35** | 8.45 | 6.24 | 0.01 | 0.50 | **0.33** | 2.59 | 0.61 | **1.08** | 0.54 |
| AIC | **-18.71** | -14.90 | -10.48 | 1.98 | 1.01 | **1.34** | -3.19 | 0.78 | **-0.17** | 0.91 |
| BIC | **-14.02** | **-10.21** | -5.78 | 6.67 | 5.70 | **6.03** | 1.50 | 5.47 | **4.52** | 5.60 |
| **Student-t** | | | | | | | | | | |
| LL | 9.45 | **10.34** | **8.72** | -1.65 | 0.78 | -1.67 | 4.43 | -0.10 | 0.72 | **1.49** |
| AIC | -14.90 | **-16.68** | **-13.43** | 7.29 | 2.45 | 7.34 | -4.85 | 4.20 | 2.56 | 1.02 |
| BIC | -5.52 | -7.30 | -4.05 | 16.67 | 11.83 | 16.72 | 4.53 | 13.58 | 11.94 | 10.40 |
| **Clayton** | | | | | | | | | | |
| LL | 1.72 | 6.1 | 3.45 | -0.07 | **1.43** | -0.99 | -2.78 | **1.73** | 0.17 | 0.36 |
| AIC | -1.43 | -10.2 | -4.91 | 2.15 | **-0.86** | 3.98 | 7.57 | **-1.45** | 1.66 | 1.28 |
| BIC | 3.26 | -5.52 | -0.22 | 6.84 | **3.83** | 8.67 | 12.26 | **3.24** | 6.35 | 5.97 |
| **Rotated Clayton** | | | | | | | | | | |
| Degree | 90 | 270 | 270 | 180 | 180 | 180 | 180 | 270 | 180 | 180 |
| LL | 10.2 | 6.66 | 7.07 | **0.14** | 0.08 | -0.37 | **5.59** | 0.79 | 0.94 | 0.73 |
| AIC | -18.3 | -11.3 | -12.1 | **1.72** | 1.84 | 2.75 | **-9.18** | 0.42 | 0.13 | **0.54** |
| BIC | -13.6 | -0.11 | **-7.44** | **6.41** | 6.53 | 7.44 | **-4.48** | 5.11 | 4.82 | **5.23** |

Note: The log-likelihood, AIC and BIC value are reported. The best-fitted copula is selected based on the AIC, BIC, and log-likelihood value.

Table C3. Constant copula estimation results for the long run.

| **-** | **BDI** | **BDTI** | **SHCI** | **SP500** | **MSCI_E** | **USD** | **Gold** | **SP_Agri** | **EUA** | **GPRD** |
| --- | --- | --- | --- | --- | --- | --- | --- | --- | --- | --- |
| **Gaussian** | | | | | | | | | | |
| LL | 54.97 | 0.23 | 0.81 | 4.13 | 1.02 | 0.05 | 0.86 | 0.10 | 3.95 | 3.40 |
| AIC | -107.93 | 1.55 | 0.38 | -6.26 | -0.05 | -18.09 | 0.29 | 1.81 | -5.90 | -4.79 |
| BIC | -103.24 | 6.24 | 5.07 | **-1.57** | 4.64 | -13.40 | 4.98 | 6.50 | -1.21 | -0.10 |
| **Student-t** | | | | | | | | | | |
| LL | 50.45 | 0.92 | **4.36** | **7.23** | **5.89** | 10.95 | -0.86 | **4.20** | 1.96 | **7.03** |
| AIC | -96.91 | 2.16 | **-4.71** | **-10.46** | **-7.77** | -17.90 | 5.72 | **-4.40** | 0.08 | **-10.05** |
| BIC | -87.52 | 11.54 | 4.67 | -1.08 | 1.61 | -8.52 | 15.10 | **4.98** | 9.46 | -0.67 |
| **Clayton** | | | | | | | | | | |
| LL | 33.52 | -0.69 | 1.92 | -8.38 | 2.90 | 2.32 | **3.30** | 0.14 | -7.12 | -1.61 |
| AIC | -65.05 | 3.38 | -1.84 | 18.78 | -3.80 | -2.64 | **-4.61** | 1.72 | 16.24 | 5.22 |
| BIC | -60.35 | 8.07 | **2.85** | 23.47 | 0.88 | 2.04 | **0.08** | 6.41 | 20.93 | 9.91 |
| **Rotated Clayton** | | | | | | | | | | |
| Degree | 90 | 180 | 180 | 270 | 270 | 180 | 180 | 90 | 180 | 90 |
| LL | **58.64** | **1.76** | 0.49 | 2.81 | 3.36 | **18.7** | -4.35 | 0.82 | **6.73** | 4.26 |
| AIC | **-115.28** | **-1.52** | 1.02 | -3.63 | -4.72 | **-35.5** | 10.70 | 0.37 | **-11.46** | -6.53 |
| BIC | **-110.59** | **3.16** | 5.71 | 1.06 | **-0.02** | **-30.8** | 15.39 | 5.06 | **-6.76** | **-1.84** |

Note: The log-likelihood, AIC and BIC value are reported. The best-fitted copula is selected based on the AIC, BIC, and log-likelihood value.
