# Supplementary material for: Identifying the significant drivers of containerized freight rates: From the perspective of dynamic multiscale dependence
Source: PLoS One. 2026 Apr 21;21(4):e0344386. doi: 10.1371/journal.pone.0344386 (PMC13099089; doi:10.1371/journal.pone.0344386)
Supplement: Attachment — Submitted filename: Response to reviewers R2.docx [file pone.0344386.s005.docx]

Reviewer #2:

**Thank you very much for your useful comments. In this version, we revised our paper according to your suggestions. In the revised manuscript, we highlight the places that have been modified. Please refer to our revised version. Point-by-point responses are listed below.**

1. In the abstract, what phenomenon of the Shanghai Containerized Freight Index (SCFI) futures highlights the growing demand for risk management in the container shipping market?

**Answer:** According to the original intention of the paper, we modified the first sentence of the abstract. Please refer to the highlight words.

2. In the abstract, the research on the factors behind container freight rate fluctuations is still limited. How is this reflected?

**Answer:**  Refer to this issue, we have pointed out in the literature review in Section 1. So far, most researches emphasized on the impact factors of dry bulk freight rates. The researches on container freight rates focus on the prediction method design. Please refer to the highlight sentences in Section 1.

3. In the abstract, this paper provides important reference significance for various stakeholders with different financial attributes in activities such as trading and risk management, covering a wide time frame. This can be outlined in the abstract.

**Answer:** Due to the limited word count of the abstract, we were unable to fully express the suggestions in the abstract. The detailed conclusions and suggestions are in Section 4.4 and the second paragraph of Section 5, respectively. Please refer to the highlight sentences. In the meanwhile, we elucidate the significance briefly in the abstract.

4. The article contains colloquial expressions, and I suggest proofreading the entire text.

**Answer:** Thank you for your suggestion. We proofread the entire text in a further step.

5. The paper conducts extensive analysis from the perspective of dynamic multiscale dependencies to identify the key driving factors of container freight rates. What is the practical value of the core results in guiding practice?

**Answer:** we pointed out the practical implications in 4.4. These suggestions are aimed at enterprises related to container freight assets. These enterprises include shipping companies with container capacity, shipper companies that require container capacity, and non-shipping institutions participating in Shanghai Containerized Freight Index futures trading. These implications help them in clarifying the different tail risk associations between container freight assets and other assets at different time scales. The third author of this article is currently employed at the Shanghai Futures Exchange, and he believes that our research is also beneficial for the promotion of Shanghai Containerized Freight Index futures. The conclusion of this article is helpful for traders of other financial assets to consider this future when constructing investment portfolios.

6. The research contribution of this study repeatedly emphasizes that certain research is being conducted for the first time. Is this valid? Is there sufficient support for this claim? It is suggested to elaborate on this aspect.

**Answer:** We reviewed the related research articles and found no scholars have conducted similar research before. We also pointed out the characteristics of some related research in the fourth paragraph of Section 1. To be more rigorous, we have removed the two “first” in the abstract and conclusion in the revised version. We hope you give us a chance to publish.

Moreover, the first author has studied shipping freight rates for more than ten years. She has been following the trend of shipping rates and the development of the shipping rate derivatives market. She also published several papers on forecasting container freight rates, identifying the risk spillovers between shipping freight rates and other factors. She found there were indeed no articles before that were similar to this one in terms of analysis methods and asset scope.

7. Is the necessary validation of the model analysis complete? It is recommended to demonstrate the feasibility of the model from different perspectives.

**Answer:** We think the model analysis is completed. The research idea of this article is as follows. Firstly, EMD is a typical multiscale analysis, which has been widely used in analyzing the internal and latent data-generating process in both time and frequency domains. MEMD, used in this research, is an extension of EMD in multivariate data processing. MEMD can decompose multiple variables within a unified framework, thus enabling the comparability analysis of different variables in different time domains in this article. However, due to the large number of IMFs obtained by MEMD, we reorganized the IMFs based on their entropy values. Secondly, this paper used LASSO to identify the key influencing factors in different time domains. We also analyze the results in detail, please refer to Section 4.2. Finally, we use copula to analyze the tail dependence between SCFI and the factors in different time domains. Especially, we use dynamic copula to investigate the time-varying tail-dependency. We also provide a research flowchart, please refer to Figure 1.

Refer to the method selection, the EMD family methods for decomposing data in time-domain has been employed in numerous articles on time series analysis, such as Zhang and Zeng(2017), Zhu et al. (2019). LASSO has been found to effectively identify influential variables and those that can offer additional valuable information, please refer to Zhang et al.(2019). Copula is a common method for characterizing the tail risk dependence of financial assets. In recent years, many researchers have applied themselves to extending and applying the copula method in shipping markets, such as Bai(2021), Zhang(2018).

In a short summary, identifying the key factors of SCFI and investigating the tail-dependency between SCFI and other financial assets in time-domain is our research target. We serve this goal in both the research process and the selection of research methods. We elaborate our research idea in the paragraph above Figure 1.

**References:**

[1]. Zhang, Jiao , and Q. Zeng . "Modelling the volatility of the tanker freight market based on improved empirical mode decomposition." Applied Economics 49.17(2017):1-13.
[2]. Bangzhu Zhu, Shunxin Ye et al. "A multiscale analysis for carbon price drivers." Energy Economics 78(2019):202-216.
[3]. Zhang, Yaojie , F. Ma , and Y. Wang . "Forecasting crude oil prices with a large set of predictors: Can LASSO select powerful predictors?." Journal of Empirical Finance 54(2019).
[4]. Bai X. Tanker freight rates and economic policy uncertainty: A wavelet-based copula approach." Energy (2021).
[5]. Zhang, Yi . "Investigating dependencies among oil price and tanker market variables by copula-based multivariate models." Energy (2018): 435-446.

**When you read my responses, it may be the beginning of the new year. On behalf of all the authors of this article, I wish you all the best in 2026!**

**Reviewer #3: The revised version shows substantial improvements in structure, clarity, and methodological justification.**

**When you read my responses, it may be the beginning of the new year. On behalf of all the authors of this article, I wish you all the best in 2026!**

**Reviewer #4:**

**Thank you very much for your insightful comments. In this version, we revised our paper according to your suggestions. In the revised manuscript, we highlight the places that have been modified. Point-by-point responses are listed below.**

• Oscillatory-based nonlinear mode decomposition, which is well established in dynamical systems theory, and I believe has the potential for both more granular (in time) and accurate time-series decompositions (Iatsenko, McClintock, Stefanovska, Physical Review E 2015).

**Answer:** Thank you for the methods you recommended. Due to our limited knowledge, we didn’t know this method. Therefore, we mentioned at the end of the article that there are shortcomings in the selection of decomposition methods. Please refer the highlight sentences in the last paragraph.

The related article indicates the necessary MATLAB codes for running NMD are freely available for download, which definitely facilitates the social science researchers to apply this method. In the future, we will consider to use nonlinear mode decomposition in our research.

• That while the complexity of financial market dynamics makes their modelling as stochastic (e.g. by copula) not unreasonable, market interactions are fundamentally deterministic and application of stochastic methods only is insensitive to the underlying determinism (Rowland Adams, Newman, Stefanovska, EPSJ:ST 2023). There are methods from multiscale nonlinear systems theory that are designed for application to this kind of system, and could give highly informative results, in addition to the copula analysis (https://ui.adsabs.harvard.edu/abs/2019zndo...3470856I/abstract, User Manual).

**Answer: Because we can’t open the URL directly,** we think you may indicate this article “An order parameter for synchronisation of angular velocities”. But this article contains a lot of mathematical and physical knowledge, which makes it a bit difficult for us to study in one month. Due to limited time for revision, we apologize that we are currently unable to understand the connection between this article and our manuscript. We will pay attention to the applications of this method proposed in this article in the future, and look forward to apply it in shipping freight rates analysis in the near future.

However, I repeat that the authors’ findings are interesting and publishable in their own right, and that application of the above methods is more appropriate for a separate contribution than inclusion in this current one at this stage.

**Answer:** Thank you for this comment!

**When you read my responses, it may be the beginning of the new year. On behalf of all the authors of this article, I wish you all the best in 2026!**

Reviewer #5:

**Thanks for your comments and suggestions. In this version, we revised our paper according to your suggestions.**

• I recommend a final round of language polishing. Examples:

1. “skewd student-t” → “skewed Student-t” in the EGARCH description.

2. Missing spaces around some symbols, e.g. “λis the adjustment parameter” → “λ is the adjustment parameter.”

**Answer:** We fixed these issues and proofread the whole article carefully to avoid any typos and grammar mistakes.

**When you read my responses, it may be the beginning of the new year. On behalf of all the authors of this article, I wish you all the best in 2026!**
